# Fundamental changes in regulation to improve access to biosimilars

**DOI:** 10.2471/BLT.25.294908

**Published:** 2026-05-11

**Authors:** Hye-Na Kang, Ivana Knezevic, Meenu Wadhwa, Eun Kyung Kim

**Affiliations:** a Medicines and Health Products Policies and Standards Department, World Health Organization, Avenue Appia 20, 1211 Geneva 27, Switzerland.; b Biotherapeutics and Advanced Therapies, R&D, Medicines and Healthcare products Regulatory Agency, Potters Bar, England.

Biotherapeutic products such as monoclonal antibodies, insulins and erythropoietins have revolutionized the treatment of a wide range of diseases, from cancer and autoimmune disorders to chronic diseases. However, the high prices, reflecting the complexity and cost of development and manufacturing, pose a considerable challenge to health-care sustainability and patient access worldwide. In response, a biosimilar, that is, a biological product shown to be highly similar in terms of its quality, safety and efficacy to an already licensed reference product, has emerged as a critical solution to promote competition and reduce prices.^1^

The World Health Organization (WHO) has provided global leadership on biosimilar regulation for many years. The first WHO *Guidelines on evaluation of biosimilars* issued in 2009 offered a scientific framework for countries developing regulatory pathways for biosimilars. In 2014, the World Health Assembly adopted Resolution WHA67.21, calling for improved access to quality-assured biotherapeutics, including biosimilars.^2^ The 2022 revision of the WHO guidelines^1^ reflects more than a decade of regulatory experience and scientific advances that support more efficient, risk-based evaluation.^3^^,^^4^ The revision introduces important regulatory flexibilities that allow a streamlined clinical evaluation approach whereby a clinical efficacy study is not required to demonstrate biosimilarity if certain criteria are met and biosimilarity can be established based on the totality of evidence (that is, when all available data are considered together to reach a decision). An increased emphasis on quality and functional in vitro assessment enables reduction in cost and development timelines, and supports swifter regulatory approval as a critical first step towards product availability. Consequently, this shift would allow for other measures, such as improving affordability for broader access to biotherapeutics, including biosimilars, especially in low- and middle-income countries where access is limited.

Although detailed technical guidance on evaluation of biosimilars already exists, many national regulatory authorities and policy-makers continue to face practical difficulties in interpreting and applying these principles. This situation is due to challenges in translating scientifically complex, risk-based evaluation principles into regulatory decision-making processes within technical and resource-constrained settings. Lack of expertise and uncertainty in applying flexibility within legal and policy frameworks are additional difficulties. This article addresses the gap by translating the scientific concepts of the revised guidelines into clear, policy-oriented messages, highlighting opportunities, risks and implementation considerations for regulators, particularly in low- and middle-income countries. Adoption of practical and pragmatic solutions can assist in timely and equitable product access to patients.

## An overview of biosimilars

The global biosimilars market has expanded and continues to play a vital role in improving access to essential biotherapeutics. As of 2025, biosimilars represent a multibillion-dollar industry, with the European Union (EU) and United States of America leading in approvals and market penetration. The economic impact of biosimilars is substantial. Since 2015, biosimilars have generated an estimated 36 billion United States dollars (US$) in cumulative savings in the United States, including US$ 12.4 billion in 2023 alone, indicating an increase in savings over time.^5^ A 2022 study further projected biosimilar savings of US$ 38.4–124.5 billion for 2021–2025.^6^ In the EU, competition from biosimilars has delivered over 56 billion euros in cumulative savings by mid-2024.^7^ Similar trends are observed in parts of the Western Pacific Region, such as Singapore, which saved an estimated US$ 136 million in just five years due to value-driven adoption strategies.^8^ These savings underscore the capacity of biosimilars to alleviate financial burdens on health systems, while simultaneously improving patient access. A diverse portfolio of biosimilar products is now available across therapeutic areas (Table 1). These benefits demonstrate that effective regulation of biosimilars is a critical component of sustainable health systems. However, in low- and middle-income countries, access to biosimilars of assured quality is limited because numerous challenges have hindered local manufacturing of biosimilars in these countries. These challenges are not only limited to the high costs of biosimilars but are also attributed to limited investment in research and development, technology transfer and public–private partnerships. Establishing well-designed public–private partnerships between health agencies, local manufacturers and global biosimilar producers can leverage advantages to develop and provide access to biosimilar products that target local public health needs. In addition, the lack of specialist knowledge and inappropriate regulatory processes for biologicals in some low- and middle-income countries are also confounding factors. In fact, mislabelled biosimilars (that is, with no demonstrated similarity with the reference product) exist in some countries. Importantly, biosimilars should be regulated according to the requirements for biologicals, as emphasized in the WHO guidelines^1^ and related documents. 

**Table 1:** Approved biosimilars in the EU, Australia and United States of America, March 2026

Product	EU	Australia	United States	WHO *2025 Model list of essential medicines*
**Peptide or hormone**				
Teriparatide^a^	Approved	Approved	NA	NA
Insulin aspart	Approved	Approved	Approved	Approved
Insulin glargine	Approved	Approved	Approved	Approved
Insulin lispro	Approved	NA	NA	Approved
Somatropin^a^	Approved	NA	NA	NA
Erythropoietin	Approved	Approved	Approved	Approved
Follitropin α	Approved	Approved	NA	NA
**Anticoagulant**				
Enoxaparin^a^	Approved	Approved	NA	Approved
**Cytokine**				
Filgrastim	Approved	Approved	Approved	Approved
Pegfilgrastim	Approved	Approved	Approved	Approved
**Fusion protein**				
Aflibercept	Approved	Approved	Approved	NA
Etanercept	Approved	Approved	Approved	Approved
**Monoclonal antibody**				
Adalimumab	Approved	Approved	Approved	Approved
Bevacizumab	Approved	Approved	Approved	Approved
Denosumab	Approved	Approved	Approved	NA
Eculizumab	Approved	NA	Approved	NA
Golimumab	Approved	NA	NA	Approved
Infliximab	Approved	Approved	Approved	Approved
Natalizumab	Approved	Approved	Approved	NA
Omalizumab	Approved	Approved	Approved	NA
Pertuzumab	Approved	NA	Approved	NA
Ranibizumab	Approved	Approved	Approved	NA
Rituximab	Approved	Approved	Approved	Approved
Tocilizumab	Approved	Approved	Approved	NA
Trastuzumab	Approved	Approved	Approved	Approved
Ustekinumab	Approved	Approved	Approved	Approved

EU: European Union; NA: not applicable.

^a^ Not on United States Food and Drug Administration Biosimilar list.

## Regulatory evaluation

Since biotherapeutics are large and complex proteins that are more challenging to characterize compared to chemically synthesized small molecules, the generic medicines approach applicable to chemical drugs is not appropriate for licensing biotherapeutics.^9^ Therefore, countries using incorrect procedures for biologicals, including biosimilars, should act swiftly and remediate this issue. Doing so is one of the critical steps for improving regulatory convergence as part of the initiative towards better access to biotherapeutic products of assured quality, safety and efficacy worldwide.

The 2022 revised WHO guidelines prioritize analytical comparability and mainly pharmacokinetics (with inclusion of pharmacodynamics markers where available) studies over mandatory large-scale clinical efficacy studies.^1^ This prioritization is consistent with evolving practices of regulatory authorities such as the United States Food and Drug Administration, European Medicines Agency, the Medicines and Healthcare products Regulatory Agency of the United Kingdom of Great Britian and Northern Ireland and Health Canada, which increasingly accept streamlined clinical trials without comparative clinical efficacy studies when robust totality of evidence is demonstrated. The shift in emphasis is towards a stronger focus on quality, supported by extensive data from analytical and functional assessments using multiple complementary methods, considering how structure relates to function and the product’s mechanism of action. This approach is scientifically based on the volume of data, knowledge and experience in assessing biosimilarity. Notably, the aim of a biosimilar evaluation is to establish the similarity of a candidate biosimilar to the reference product and not to independently establish the safety and efficacy of a candidate biosimilar.^4^ Based on proven similarity, the licensing of a biosimilar would rely partly on clinical data of the licensed reference product (that is, the originator).^1^ Data and experience over the last decade clearly show that comparative clinical efficacy and safety studies have not been critical for regulatory approval. Any differences between a candidate biosimilar and the reference product in clinical comparability were resolved through physicochemical and functional data together with comparative pharmacokinetics studies. To date, confirmatory clinical trials have played a limited role in addressing quality concerns and in revealing any additional information on the immunogenicity of biosimilars that was not inferred from analytical and pharmacokinetics studies.^10^^–^^12^ In fact, some biosimilars have been approved despite failed clinical trials.^11^ Importantly, while clinical efficacy studies may not be necessary in most cases, specific situations, albeit rare, may arise where comparative clinical efficacy, safety and/or immunogenicity data are required for ensuring similar clinical performance. As an example, a product where the mechanism of action is unknown or poorly understood would fall within this category.^12^

The revised WHO guidelines focus on a strategic shift from a uniform, prescriptive evaluation framework towards a more flexible and evidence-based approach that allows for a convincing demonstration of biosimilarity based on comprehensive quality, in vitro functional assessments and the totality of evidence.^3^ This approach is scientifically justified and offers clear benefits for both patients and the health-care system, which include reducing development costs, accelerating access and aligning global regulatory practices (regulatory convergence with scientific advances).^4^^,^^10^^–^^12^ However, the approach also introduces risks in settings with weak pharmacovigilance as well as potential challenges in public trust and perception. Regulatory flexibility does not mean lower standards. These risks can be mitigated through strengthened post-marketing surveillance, including active pharmacovigilance systems, clear product identification and traceability, and targeted risk-management plans. In addition, transparent communication and stakeholder engagement are essential to build confidence in biosimilars. Such measures are particularly important in countries where pharmacovigilance systems are still developing. Absence of pre-approval efficacy trials increases the importance of strong systems for product quality oversight, adverse-event monitoring and transparent communication with health workers and patients.

Table 2 summarizes the main advantages and potential risks of the streamlined approach and links them to practical actions required at country level. Effective regulatory assessment with integration of post-marketing surveillance, education of stakeholders on biosimilarity and reliance mechanisms^13^ are essential for successful implementation. To maximize the benefits and minimize the risks of the revised framework, countries should first align their national regulatory framework with the revised WHO approach and build capacity and expertise in advanced analytics and regulatory assessment to eventually provide interchangeable biosimilars. Strong pharmacovigilance systems are essential for monitoring safety and immunogenicity post-approval. Countries should also enable regulatory reliance on assessments from mature authorities,^14^ facilitate awareness-raising, as well as stimulate communication to build trust among health workers and patients.^9^ Finally, policy-makers should develop policies that ensure that cost savings translate into affordable access for patients through procurement and reimbursement.

**Table 2:** Advantages and risks of the streamlined approach and WHO-specific recommendations for its implementation

Points to consider	Advantages	Risks and challenges	WHO recommendations
Increasing access	Faster approval and market entry due to avoiding lengthy comparative clinical efficacy studies; accelerated patient access	Robust post-marketing pharmacovigilance systems are required to pick up rare or long-term safety signals (these are not detected in comparative clinical efficacy studies)	Apply streamlined approach on a case-by-case basis (such as insulin, simple biologics, monoclonal antibodies), with stronger pharmacovigilance for all biologicals
Increasing affordability	Cost reduction on efficacy trials, leading to more affordable biosimilars and improved market competition, especially in low- and middle-income countries where affordability is critical	Some may perceive the absence of efficacy trials as lowering standards, reducing regulatory confidence and public trust in biosimilars; without confidence, interchangeability cannot be designated or make the treatments more accessible to more patients; savings may not translate into product access without supportive policies such as educational programmes for health workers, establishing patient groups, reimbursement policies, procurement strategies, effective communication and networks	Educate regulators, health workers, and patients; support low- and middle-income countries with cost–access linkage strategies (procurement, reimbursement)
Scientific issues across products	Analytical and pharmacokinetics and/or pharmacodynamics data are more sensitive and reproducible than efficacy trials in detecting differences between the biosimilar and its reference product	One-size-fits-all approach may not be relevant in some cases. Additional data may be needed in cases where mechanistically appropriate meaningful data covering complex mechanism of action are not provided despite availability of advanced in vitro functional assays. Lack of knowledge and good understanding of structure–function relationships and/or the relevance of critical quality attributes to clinical impact. Clinical efficacy studies may be needed for complex products where mechanism of action is unknown or poorly understood or for products with significant structural heterogeneity (such as heavily glycosylated proteins) and in other scenarios^12^	The shift aligns regulatory requirements with advances in analytical science. Encourage a case-by-case scientific justification, with attention to products where immunogenicity may have clinical relevance
Ethical issues	Reduce unnecessary patient exposure to clinical trials when biosimilarity is analytically established; prevent duplicative clinical trials which add little scientific value; minimize animal testing by reducing the reliance on in vivo animal studies	Possibility of underestimated immunogenicity risks in the pre-approval evaluation	Require robust post-marketing risk management plans and global safety monitoring systems
Equity in implementation	Reduce the burden in development and regulation in low- and middle-income countries	Weak pharmacovigilance systems may fail to detect rare adverse events	WHO should continue to promote the strengthening of post-market surveillance networks and encourage reliance models across regions
Regulatory convergence and alignment	Foster a more global harmonized regulatory framework; reduce redundancy and complexity; facilitate reliance and mutual recognition across regions (convergence)	Some regulators and prescribers may have less technical expertise and experience	WHO should provide opportunities for capacity-building, training and communication tools to strengthen regulator and clinician confidence

WHO: World Health Organization.

WHO’s mandate is to establish global norms and standards and promote regulatory convergence at the global level. WHO also supports countries in implementing these standards, including alignment with a streamlined approach for biosimilar approvals, and in strengthening regulatory capacity, particularly in low- and middle-income countries. Effective adoption of the up-to-date standards requires coordinated and continued action. In this context, WHO supports Member States in: (i) implementing global standards at the country level; (ii) applying the case-by-case approach for each product class; (iii) setting up and strengthening pharmacovigilance systems (post-marketing safety monitoring); and (iv) capacity-building and transparent communication to ensure trust among regulators, clinicians and patients.

The revised WHO guidelines represent a timely paradigm shift from a prescriptive, trial-heavy model towards a modern, science-driven risk-based approach. By enabling more efficient development and approval of biosimilars without compromising quality or safety, the guidelines offer a realistic pathway to expand access to life-changing biotherapeutics. 
